# Une cryptococcose disséminée compliquant un traitement par prednisone et azathioprime d'un pemphigus vulgaire

**Published:** 2011-11-11

**Authors:** Ammouri Wafa, Harmouche Hicham, Afifi Yassir, Tazi Mezalek Zoubida, Adnaoui Mohamed, Aouni Mohamed, Hassani Amine, Maaouni Abdelaziz

**Affiliations:** 1 Service de médecine interne, CHU Avicenne, Rabat, Maroc; 2 Service de médecine interne, hôpital international Cheikh Zaid, Rabat, Maroc; 3 Service de dermatologie, hôpital international Cheikh Zaid, Rabat, Maroc

**Keywords:** cryptococcose, nodules, immunodépression, Maroc

## Abstract

L'infection à cryptocoque est une complication redoutable chez les patients traités par immunosuppresseurs et dont l’évolution peut être rapidement fatal en cas de retard diagnostic. Nous rapportons le cas d'une patiente âgée de 70 ans, ayant des antécédents de pemphigus vulgaire traité par prednisone et azathioprime et admise dans le service de médecine Interne pour des nodules sous cutanés atypiques. Le diagnostic retenu était celui d'une cryptococcose disséminée. L’évolution était rapidement fatale malgré le traitement antifongique.

## Introduction

*Cryptococcus neoformans* est un agent infectieux opportuniste qui s'observe essentiellement chez les sujets immunodéprimés (patients infectés par le VIH, patients transplantés, et les patients traités par corticoïdes ou immunosuppresseurs) [1]. La porte d'entrée de ce champignon est pulmonaire et la dissémination de l'infection se fait par voie hématogène. Les principaux sites de dissémination incluent le liquide céphalorachidien, la peau, l'os, les articulations, les reins, la rate puis la prostate [2,3].

Nous rapportons à ce propos, l'observation d'un patient suivi pour un pemphigus vulgaire et qui a présenté une infection disséminée à cryotocoque au cours du traitement par prednisone et azathioprime.

## Observation

Un patient âgé de 71 ans est admis pour une altération de l’état général. Ses antécédents sont marqués par un diabète de type 2 traité par insuline et un pemphigus vulgaire non paranéoplasique, pour lequel le patient était suivi depuis 9 mois en dermatologie et traité par prednisone et azathioprime. Le patient était suivi régulièrement en consultation et l’évolution des lésions cutanées du pemphigus était satisfaisante avec disparition des lésions cutanées et cicatrisation des bulles et de vesicules. Au 9 ème mois du traitement (sous 10mg de prednisone et 100mg/j d'azathioprime), le patient a présenté progressivement une altération de son état général avec apathie.

**Figure 1:**
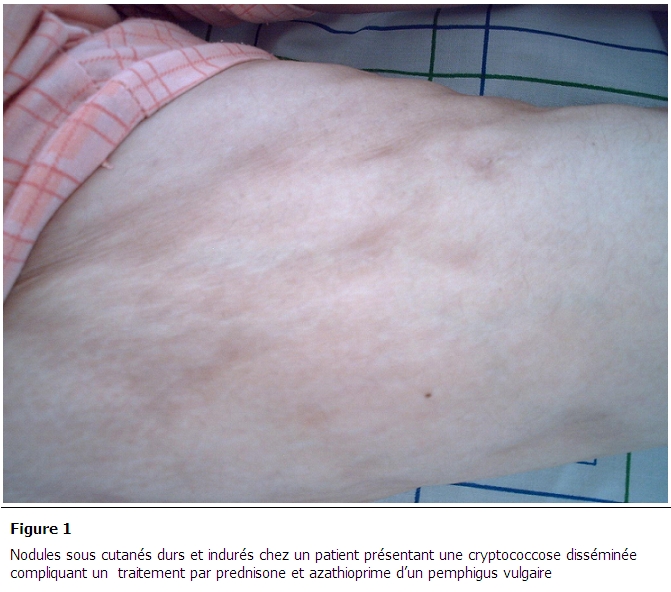
nodules sous cutanés durs et indurés chez un patient présentant une cryptococcose disséminée compliquant un traitement par prednisone et azathioprime d'un pemphigus vulgaire

A son admission, l'examen clinique a objectivé un patient apyrétique à 37°, un pouls à 90 bat/mn et une tension artérielle à 130/60 mmHg. L'examen neurologique objectivait un ralentissement psychomoteur, une désorientation temporale, la nuque était souple, les reflexes ostéo-tendineux étaient présents, les paires crâniennes étaient normales et il n'existait pas de déficit sensitif ou moteur. L'examen cutanéo-muqueux objectivait des nodules violacés au niveau du tronc, de consistance dure et douloureuse (Figure 1). L'examen cardio-vasculaire, pleuro-pulmonaire était normal et le reste de l'examen somatique n'objectivait pas d'anomalies.

Sur le plan biologique, le taux de globules blancs était à 7020/ mm^3^, les lymphocytes à 6300/mm^3^, les plaquettes à 210000/mm^3^, l'hémoglobine à 14,8g/dl, la CRP à 3mg/l, la vitesse de sédimentation à 20mm à la première heure. Le taux de prothrombine à 81%, la natrémie à 128,5mg/l, la calcémie à 89mg/l et la glycémie à 1,80g/l. la fonction rénale et hépatique étaient normales.

**Figure 2:**
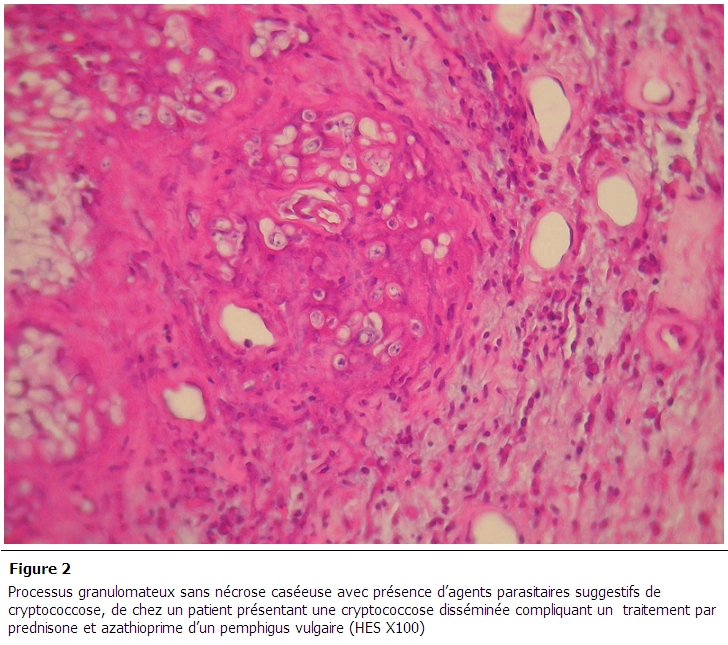
processus granulomateux sans nécrose caséeuse avec présence d'agents parasitaires suggestifs de cryptococcose, de chez un patient présentant une cryptococcose disséminée compliquant un traitement par prednisone et azathioprime d'un pemphigus vulgaire (HES X100)

L'imagerie par résonnance magnétique cérébrale était normale. La ponction lombaire a objectivée une liquide eau de roche avec une proteinorrachie à 1,89 g/l et une glycorrachie à 0,59 g/l. Le taux leucocytes dans le liquide céphalorachidien (LCR) était de 137 E/mm^3^ avec 100% de lymphocytes. L'examen direct du LCR et la culture ont isolé des colonies de *Cryprococcus neoformans*. Par ailleurs, l'examen cytobactériologique du LCR ainsi que la recherche du bacille de Koch étaient négatifs. Une biopsie cutanée a été réalisée et a objectivé la présence de Cryptocoque avec la présence d'un processus inflammatoire granulomateux sans nécrose (Figure 2). La sérologie VIH était négative. Le diagnostic d'une cryptococcose disséminée a été posé et le patient a été traité par amphotéricine (0,7mg/kg/jour) associé à la Flucytosine. L’évolution était défavorable avec une aggravation progressive de l’état de conscience du patient et son décès en soins intensifs.

## Discussion

La cryptococcose disséminée est une infection grave pouvant mettre en jeu le pronostic vital du patient. L'infection est due à *Cryptococcus neoformans*, un germe encapsulé, retrouvé chez les pigeons et autres oiseaux. La contamination de l'homme se fait par voie respiratoire, par inhalation [2]. L'infection a été initialement décrite chez les patients au stade SIDA [2], mais cette infection peut être réactivée durant l'immunodépression de l'hôte essentiellement par atteinte de l'immunité cellulaire T [4]. Notre patient avait plusieurs facteurs pouvant induire une immunodépression profonde. Le diabète, le traitement par corticoïde et azathioprime ont participé à une réactivation de l'infection à Cryptocoque et à une dissémination plus importante méningée et cutanée. La manifestation la plus commune de cette infection fongique est l'atteinte du système nerveux central sous la forme d'une méningite sub-aigue ou chronique ou de cryptococcomes cérébraux dans des cas plus rares [4,5]. Les principales manifestations cliniques en cas d'atteinte méningée sont représentées par des signes méningés, une confusion, des convulsions, une altération de la vision et rarement un déficit focal. La ponction lombaire est très utile pour le diagnostic initial. Comme dans le cas de notre patient, l'analyse du LCR, montre une élévation de la pression du LCR, une leucocytose modérée, une hypoglucorrachie et une hyperproteinorrachie. La coloration à l'encre de Chine permet de confirmer la présence du champignon [2]. L'atteinte parenchymateuse cérébrale s'observe sous forme de cryptococcomes, d'une dilatation des espaces de Virchow ou de nodules corticaux. L'IRM est l'examen de choix pour ce type de lésions, elle montre des lésions hypointense en T1 et hyperintense en T2 sans prise de contraste [1,4,5]. Notre observation démontre que l'infection disséminée à Cryptocoque touchant le système nerveux central peut s'accompagner aussi bien de manifestations neurologiques que cutanées. L'atteinte cutanée s'observe dans 10 à 20% des cas des cryptococcoses disséminées et les lésions rapportées se présentent sous forme de papules, nodules, vésicules, ulcérations, ecchymoses ou abcès. Ces lésions sont non spécifiques et peuvent être confondues avec des lésions de kaposi, un carcinome baso-cellulaire, une cellulite, des lésions acnéiforme ou varicelleuses, un molluscum contagiosum et le pyoderma gangrenosum [7,8]. La biopsie cutanée et la culture permettent d'avoir un diagnostic définitif [7].

La cryptococcose disséminée constitue une urgence thérapeutique, nécessitant un traitement antifongique agressif. Le traitement standard est à base d'amphotericine B (0,7-1mg/kg/jour) associé à la flucytosine [2,6].

## Conclusion

Ce cas clinique illustre la difficulté du diagnostic et de la prise en charge des patients ayant une cryptococcose disséminée. Notre patient n'avait aucun signe méningé et les lésions cutanées n’étaient pas spécifiques. Cette infection mérite d’être évoquée par le clinicien devant toute lésion cutanée inexpliquée, trouble cognitif ou symptôme neurologique focal survenant chez un patient immunodéprimé.
